# Nanowire morphology control in Sb metal-derived antimony selenide photocathodes for solar water splitting†

**DOI:** 10.1039/d4ta07389d

**Published:** 2025-02-17

**Authors:** Zhenbin Wang, Yongping Gan, Erin Service, Pardis Adams, Thomas Moehl, Wenzhe Niu, S. David Tilley

**Affiliations:** a Department of Chemistry, University of Zurich Winterthurerstrasse 190 8057 Zurich Switzerland wenzhe.niu@epfl.ch david.tilley@chem.uzh.ch; b Laboratory of Photonics and Interfaces, Institute of Chemical Sciences and Engineering, École Polytechnique Fédérale de Lausanne Lausanne 1015 Switzerland

## Abstract

We report a facile method to enhance the photoelectrochemical (PEC) performance of Sb_2_Se_3_ photocathodes by controlling the growth of bilayer Sb_2_Se_3_ consisting of vertically oriented nanorods on a compact Sb_2_Se_3_ layer. Sb_2_Se_3_ thin films with controllable nanorod diameters were achieved by manipulating the substrate temperatures during metallic Sb thin film deposition. The lower temperature-derived Sb_2_Se_3_ photocathode, with a larger nanorod diameter (202 ± 48 nm), demonstrated a photocurrent density of −15.2 mA cm^−2^ at 0 V_RHE_ and an onset potential of 0.21 V_RHE_. In contrast, the higher temperature-derived Sb_2_Se_3_ photocathode, with a smaller nanorod diameter (124 ± 28 nm), exhibited an improved photocurrent density of −22.1 mA cm^−2^ at 0 V_RHE_ and an onset potential of 0.31 V_RHE_. The enhanced PEC performance is attributed to reduced charge recombination, facilitated by a shorter charge transport path in the [*hk*0] direction. This study highlights the significance of morphology control in optimizing Sb_2_Se_3_ photocathodes, providing insights for future material and device design.

## Abstract 2

Sb_2_Se_3_ photocathode performance improves by tuning substrate temperature during Sb metal deposition. Thinner nanorods result in reduced charge recombination, enhancing photocurrent by restricting charge transport in the *hk*0 direction.

## Graphical abstract

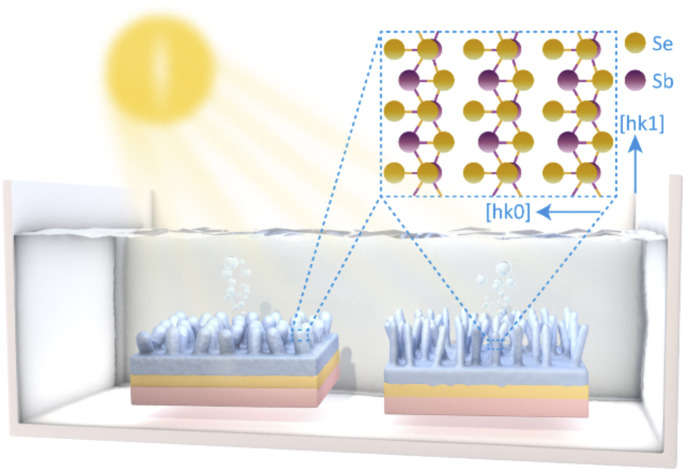

## 1. Introduction

The carbon dioxide (CO_2_) emission produced from fossil fuel combustion has been identified as a primary contributor to global warming. Therefore, developing efficient methods for generating renewable and clean energy while eliminating CO_2_ emissions is imperative.^1^ Photoelectrochemical (PEC) water splitting is regarded as a promising strategy for converting intermittent sunlight into carbon-free hydrogen fuel.^2^ In PEC systems, semiconducting materials are employed as photoelectrodes, absorbing photons to generate electron–hole pairs, thereby driving the hydrogen evolution reaction (HER) and oxygen evolution reaction (OER). Over the past few decades, various semiconducting materials have been reported for PEC water splitting, such as silicon (Si),^3^ cadmium telluride (CdTe),^4^ copper indium gallium selenide (CIGS),^5^ Cu-based chalcogenides,^6^ cuprous oxide (Cu_2_O),^7^ bismuth vanadate (BiVO_4_),^8^ and hematite (α-Fe_2_O_3_).^9^ Nonetheless, challenges such as the complex fabrication process of Si, the scarcity of In and Te, the toxicity of Cd, and the poor stability of Cu_2_O for the abovementioned materials have severely restricted large-scale commercialization. Therefore, the development of high-efficiency, low-cost, and stable semiconducting materials used in PEC systems plays a crucial role in meeting the entire society's energy demand.

Antimony chalcogenides, including Sb_2_Se_3_, Sb_2_S_3_, and Sb_2_(S, Se)_3_, have received considerable attention as promising light-absorbing materials for both photovoltaic (PV) and PEC applications, owing to their high absorption coefficient (>10^5^ cm^−1^ in the visible region), limited toxicity, tunable bandgaps (1.1–1.7 eV), and abundant availability.^10–13^ These semiconducting materials exhibit a unique one-dimensional (1D) crystal structure that facilitates efficient charge transport of photogenerated carriers along the (Sb_4_S_*x*_Se_6−*x*_) ribbon direction.^14^ Recent advancements have seen record power conversion efficiencies (PCE) for Sb_2_Se_3_ and Sb_2_(S, Se)_3_ solar cells prepared by solution-based methods, reaching 10.57% and 10.75%, respectively.^15,16^ Sb_2_Se_3_, in particular, has a proper bandgap of 1.1–1.3 eV, making it ideal for bottom cells in dual light absorber tandem cells.^17^ Its anisotropic properties result in varying hole mobilities of 1.17, 0.69, and 2.59 cm^2^ V^−1^ s^−1^ along the [100], [010], and [001] directions, respectively.^18^ However, achieving Sb_2_Se_3_ films with preferential orientation is challenging, as the (*hk*0) planes, with lower surface energy, are thermodynamically stable.^19^ Significant efforts have been made to optimize crystal orientation and minimize defects in Sb_2_Se_3_ films to enhance performance. Various methods for the preparation of Sb_2_Se_3_ have been explored in recent years, including thermal evaporation,^20^ closed-spaced sublimation,^21^ chemical bath deposition,^15^ sputtering,^22^ spin coating,^23^ and vapor transport deposition.^24^ Significant progress has been achieved using Sb_2_Se_3_ absorbers in PV cells and PEC water splitting since 2014. For example, a highly [001]-oriented Sb_2_Se_3_ photocathode was successfully obtained by extending the selenization time within a sealed quartz tube, delivering a noteworthy photocurrent density of −20.5 mA cm^−2^ at 0 V *versus* reversible hydrogen electrode (*V*_RHE_).^25^ Post-annealing and post-selenization treatments have been employed to improve crystallinity and address selenium deficiency, respectively.^26–28^ The Moon group successfully prepared bilayer Sb_2_Se_3_ using two different molecular inks through a spin-coating method, achieving a photocurrent density of 30 mA cm^−2^ at 0 V_RHE_.^29^ Additionally, Sb_2_Se_3_ nanowires with preferential orientation were synthesized using a close-spaced sublimation technique, delivering a remarkable power conversion efficiency (PCE) of 9.2%.^30^ The nanowire structure of Sb_2_Se_3_ with preferential orientation enhances sunlight absorption and facilitates efficient charge carrier extraction. Other studies have demonstrated that the substrate temperature during the deposition process significantly influences the morphology and orientation of Sb_2_Se_3_ thin films.^31^ In the meantime, our previous work indicated that flat Sb_2_Se_3_ thin films could be obtained through the selenization of electrodeposited metallic Sb thin films.^32^ Building on these insights, we hypothesized that the morphology of Sb_2_Se_3_ thin films is influenced not only by the selenization conditions but also by the properties of the metallic Sb thin film.

In this study, we investigated the impact of metallic Sb properties on the synthesis of Sb_2_Se_3_ films. By manipulating the substrate temperature during thermal evaporation, the crystal orientation of metallic Sb can be controlled. Sb thin films deposited at 25 °C and 75 °C are designated as Sb-L and Sb-H, respectively. Higher deposition temperatures lead to the preferential exposure of crystal planes with higher surface energy, significantly influencing the morphological evolution of Sb_2_Se_3_ during selenization process. Accordingly, we denote the as-prepared Sb_2_Se_3_ thin films obtained from selenization of Sb-L and Sb-H films as Sb_2_Se_3_-L and Sb_2_Se_3_-H, respectively. The champion Sb_2_Se_3_-H photocathode, comprising thinner nanorods in the FTO/Au/Sb_2_Se_3_/TiO_2_/Pt configuration, achieved a remarkable photocurrent density of −22.1 mA cm^−2^ at 0 V_RHE_ and an onset potential of 0.31 V_RHE_. In contrast, the champion Sb_2_Se_3_-L photocathode with thicker nanorods exhibited a photocurrent density of −15.2 mA cm^−2^ at 0 V_RHE_ and an onset potential of 0.21 V_RHE_. Moreover, the Sb_2_Se_3_-H photocathode demonstrated a lower dark current and a superior fill factor compared to the Sb_2_Se_3_-L photocathode. This straightforward enhancement in the PEC performance of the Sb_2_Se_3_ photocathode, achieved through control of the metallic Sb films, represents a novel approach to synthesizing Sb_2_Se_3_ films.

## 2. Results and discussion

**Fig. 1:**
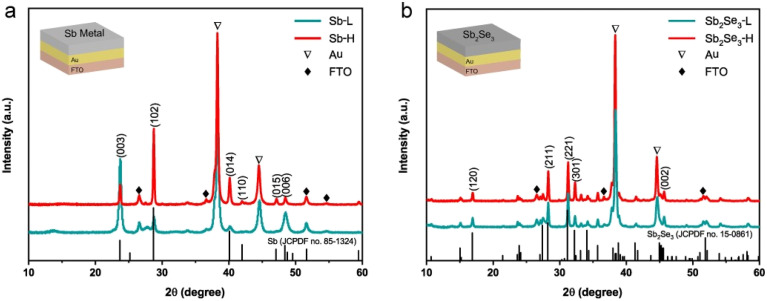
(a) XRD pattern of Sb metal deposited at substrate temperatures of 25 °C and 75 °C. (b) XRD pattern of Sb_2_Se_3_ synthesized at 325 °C for 30 min by selenizing the corresponding Sb metal sample. The configuration of the samples used for XRD measurement is provided in the inset.

A schematic illustration outlining the synthesis procedure of nanostructured Sb_2_Se_3_ thin films is depicted in Fig. S1.† 200 nm of Au layer was deposited onto FTO to form an ohmic contact with the Sb_2_Se_3_ thin films. Subsequently, a metallic Sb layer was deposited at different substrate temperatures using thermal evaporation. Although higher temperatures were tested, 75 °C produced the most promising results. For comparison, we focused on 25 °C and 75 °C in the subsequent analysis. Detailed synthesis information can be found in the experimental section. Both top-view (Fig. S2†) and cross-sectional scanning electron microscopy (SEM) images (Fig. S3†) reveal a compact and pinhole-free morphology of metallic Sb thin films. The thickness of both metallic Sb layers is around 240 nm, as determined by profilometer measurement (Fig. S3c†). X-ray diffraction (XRD) was conducted to determine the effect of substrate temperature on the crystal evolution of metallic Sb thin films. As shown in Fig. 1a, except for the peaks of Au and FTO, all diffraction peaks of Sb-L and Sb-H match well with the standard diffraction pattern of the rhombohedral Sb phase (PDF no. 85-1324). The relative ratio of surface energies for (003), (102), and (014) planes is 1.91 : 2.36 : 2.36.^33^ This indicates that the Sb-L thin film, dominated by (003) and (006) planes, has lower surface energy than that of the Sb-H thin film. With the increase of the substrate temperature, the (102) and (014) diffraction peaks with higher surface energies become more prominent in the Sb-H thin film.

The Sb_2_Se_3_ thin films were synthesized by subjecting metallic Sb thin films to selenization at 325 °C for 30 min in a sealed tube furnace under Ar protection. Fig. 1b displays the XRD pattern of the as-prepared Sb_2_Se_3_ thin films, which can be well indexed to the standard orthorhombic Sb_2_Se_3_ phase (JCPDS 15-0861).^34^ Apart from the diffraction peaks of FTO and Au, all other peaks belong to the Sb_2_Se_3_ phase, indicating the formation of a single phase without secondary phase formation. In addition, both samples display a preferred [*hk*1] orientation. There is no noticeable difference observed in the XRD patterns between Sb_2_Se_3_-L and Sb_2_Se_3_-H thin films, implying that the orientation of metallic Sb films has a negligible impact on the crystallographic orientation of Sb_2_Se_3_ thin films. Researchers have successfully prepared numerous [*hk*1]-oriented Sb_2_Se_3_ thin films through the postselenization of metallic Sb thin films at various temperatures. These Sb thin films, with initially unknown crystal orientations, were produced using diverse methods such as electrodeposition,^32^ sputtering,^22^ and e-beam evaporation.^35^ They found that the crystal orientation evolution of Sb_2_Se_3_ thin films during postselenization is primarily governed by selenization kinetics rather than the pristine crystal orientation of the Sb thin films. During the selenization process, Se atoms reach the surface of the Sb thin film and rather selenize the surface than diffuse deeper into the Sb film.^36^ This results in the formation of (Sb_4_Se_6_)_*n*_ ribbons, allowing continuous selenization along the [001] direction and promoting the perpendicular growth of Sb_2_Se_3_ films on the substrate. This finding explains the formation of identical crystal orientations in Sb_2_Se_3_-L and Sb_2_Se_3_-H thin films, despite the different exposed surfaces of their Sb precursors.

**Fig. 2:**
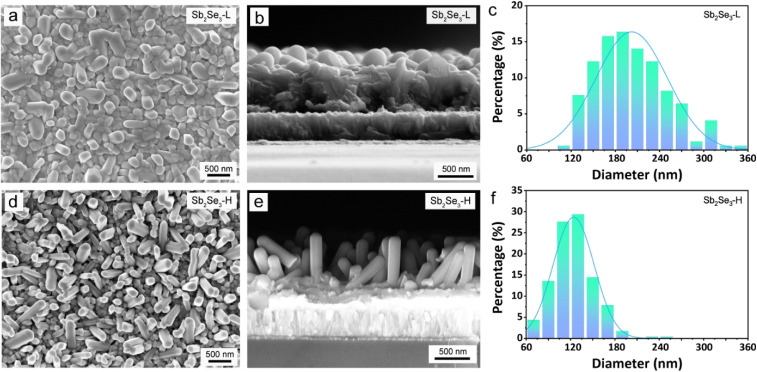
Top view (a and d) and cross-sectional view (b and e) SEM images of the (a and b) Sb_2_Se_3_-L and (d and e) Sb_2_Se_3_-H thin films. Columnar plots of the characteristic nanorod diameters of the (c) Sb_2_Se_3_-L and (f) Sb_2_Se_3_-H thin films.

Nevertheless, the morphology differs between the Sb_2_Se_3_-L and the Sb_2_Se_3_-H thin films. Fig. 2 presents the top-view and corresponding cross-sectional SEM images of the Sb_2_Se_3_-L and Sb_2_Se_3_-H thin films, indicating the formation of a bilayer structure composed of vertically oriented nanorods on top of a compact layer in both samples. The thickness of the Sb_2_Se_3_-L and Sb_2_Se_3_-H thin films was determined using profilometer measurement (Fig. S4†), revealing that the thickness of the bottom compact layer for both samples was identical, around 540 nm. The length of vertically oriented nanorods in the Sb_2_Se_3_-H thin film is longer than that of the Sb_2_Se_3_-L thin film, which is in good agreement with observations in the cross-sectional SEM images (Fig. 2b and e). However, the Sb_2_Se_3_-H thin film exhibits a denser array of nanorods compared to its counterpart, as evident from the top-view SEM images. For the Sb_2_Se_3_-H thin film, the diameter of the vertical nanorods is smaller compared with that of the Sb_2_Se_3_-L thin film. By analyzing more than 200 typical nanorods from the top-view SEM images, the statistical distributions of the diameter of the Sb_2_Se_3_ nanorods were obtained (Fig. 2c and f). The mean diameter values for the Sb_2_Se_3_-L and Sb_2_Se_3_-H thin films are 202 ± 48 and 124 ± 28 nm, respectively. Such a considerable difference in the morphology of vertically oriented Sb_2_Se_3_ nanorods can be attributed to the surface energy-driven growth during the selenization process. The as-deposited metallic Sb-H film, exposing planes with higher surface energies, preferentially reacts with Se atoms to lower their surface energy, forming a thermodynamically stable phase. Therefore, a faster rate of nucleation is expected for the Sb-H thin film, giving rise to more nanorods that are relatively thinner and longer compared to the case with a lower rate of nucleation, where the Sb_2_Se_3_ has more time to grow laterally (*i.e.*, become thicker) before encountering a neighboring rod.

X-ray photoelectron spectroscopy (XPS) measurements were further carried out to characterize the chemical state of both Sb_2_Se_3_ thin films. Fig. S5† shows XPS spectra of the Sb 3d core levels for the Sb_2_Se_3_-L and Sb_2_Se_3_-H surfaces, exhibiting identical results. The binding energies of 529.1 and 538.5 eV associated with Sb_5/2_ and Sb_3/2_ peaks were indexed to the oxidation state of Sb^3+^ in Sb_2_Se_3_, confirming the formation of Sb_2_Se_3_. Peaks located at 529.7 and 539.1 eV were attributed to Sb_5/2_ and Sb_3/2_ of Sb_2_O_3_. A small oxygen peak at 532.2 eV belonging to O 1s further confirmed the existence of Sb_2_O_3_, which originated from the selenization process due to residual O_2_ in the tube.^37^ The formation of Sb_2_O_3_ is more favorable in the presence of O_2_ because of the large Gibbs free energy (−605 kJ mol^−1^) and standard molar reaction enthalpy (−718 kJ mol^−1^).^38^ Considering that no distinguishable peak from Sb_2_O_3_ can be observed in the XRD pattern, we assumed that Sb_2_O_3_ only existed on the film's surface, in accordance with previous literature.^39^

**Fig. 3:**
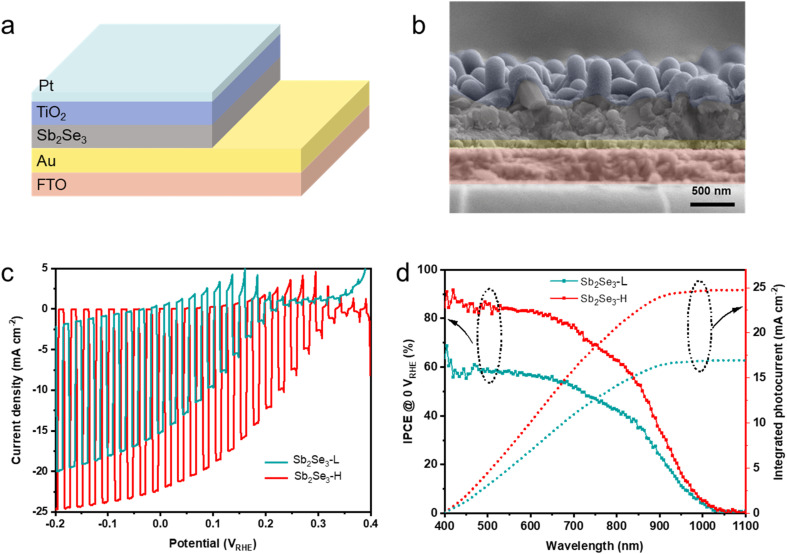
(a) Schematic illustration of the FTO/Au/Sb_2_Se_3_/TiO_2_/Pt device structure. (b) Cross-sectional false-color SEM image of a representative Sb_2_Se_3_-H device. (c) LSV measurements of the Sb_2_Se_3_-L and Sb_2_Se_3_-H photocathodes under intermittent illumination (simulated AM 1.5 G, 100 mW cm^−2^) in a 1 M H_2_SO_4_ electrolyte with a scan rate of 10 mV s^−1^. (d) IPCE and integrated photocurrent of the Sb_2_Se_3_-L and Sb_2_Se_3_-H photocathodes biased at 0 V_RHE_ in a 1 M H_2_SO_4_ electrolyte under 10% white light illumination.

To evaluate the PEC performance of the Sb_2_Se_3_ photocathodes at the device level, an n-type TiO_2_ layer was coated on the surface of the Sb_2_Se_3_*via* atomic layer deposition (ALD), which served as an electron selective contact. A nominal 2 nm Pt layer, acting as a hydrogen evolution catalyst, was deposited by sputtering. The device structure of FTO/Au/Sb_2_Se_3_/TiO_2_/Pt is depicted in Fig. 3a, and the cross-sectional SEM image of the complete device is shown in Fig. 3b. Fig. S6 and S7† reveal a thin TiO_2_ (∼50 nm) layer uniformly covering both nanostructured Sb_2_Se_3_ samples, regardless of their nanostructured morphology. The PEC performance of the Sb_2_Se_3_ photocathodes was evaluated with an Ag/AgCl (in 3 M KCl) reference electrode in a 3-electrodes electrochemical cell. The stability and reliability of the Ag/AgCl electrode was comparable to a Hg/HgSO_4_ reference electrode in strong acid solution (Fig. S8†). Fig. 3c and S9† illustrated the *J*–*V* curves of Sb_2_Se_3_ photocathodes, prepared at different substrate temperatures (25, 50, 75, and 100 °C), in a 1 M H_2_SO_4_ electrolyte (pH 0) under AM 1.5 G simulated illumination (100 mW cm^−2^). To clarify the onset potential, the *J*–*V* curves under continuous light were shown in Fig. S10.† The champion Sb_2_Se_3_-L photocathode exhibited a photocurrent density of −15.2 mA cm^−2^ at 0 V_RHE_ and an onset potential of 0.21 V_RHE_. In contrast, the champion Sb_2_Se_3_-H photocathode demonstrated a significant enhancement, with a photocurrent density of −22.1 mA cm^−2^ at 0 V_RHE_ and an onset potential shifted to 0.31 V_RHE_, outperforming the Sb_2_Se_3_-50 °C and Sb_2_Se_3_-100 °C photocathodes. This indicates that a substrate temperature of 75 °C during the Sb deposition is optimal for Sb_2_Se_3_ formation. The Sb_2_Se_3_-L photocathode exhibited a dark current at negative potentials (<−0.05 V_RHE_), whereas no dark current was observed in the Sb_2_Se_3_-H photocathode. The dark current likely originates from pinholes in the Sb_2_Se_3_ layer, which enables a direct contact of the ALD TiO_2_ layer and Pt catalyst to the back contact. The absence of a dark current in the Sb_2_Se_3_-H photocathode suggests that the optimized deposition conditions for the metallic Sb film resulted in a compact, pinhole-free Sb_2_Se_3_ layer, and is supported by the higher shunt resistance obtained from impedance spectroscopy (Table S1†). To ensure reproducibility, the statistical analysis of multiple samples confirms the same increasing trend in PEC performance across varying substrate temperatures, as shown in Fig. S11.† Additionally, the stability of the Sb_2_Se_3_-H device was recorded in a 1 M H_2_SO_4_ solution at 0.2 V_RHE_ shown in Fig. S12.† The photocurrent density remained at −10 mA cm^−2^ over 2 h and gradually decreased to −8 mA cm^−2^ after running 5 h, retaining 80% of its initial value. Researchers have reported that (NH_4_)_2_S etching treatment enhances the performance of Sb_2_Se_3_-based photocathodes and solar cells by effectively removing the Sb_2_O_3_ layer.^40,41^ XPS measurements revealed that the surface of the Sb_2_Se_3_-H sample contained more oxygen than that of the Sb_2_Se_3_-L sample. To assess the impact of oxygen on PEC performance, we conducted (NH_4_)_2_S etching treatment to Sb_2_Se_3_-H samples from the same batch to minimize variability. The PEC performance of both untreated and etched Sb_2_Se_3_ photocathodes (Fig. S13†) was identical, suggesting that the amount of oxygen does not significantly influence the PEC performance of Sb_2_Se_3_ in this study.

The incident photon-to-current conversion efficiency (IPCE) analysis was performed at 0 V_RHE_ to reveal the light-harvesting capabilities of both Sb_2_Se_3_ photocathodes in the wavelength range of 400–1100 nm, as shown in Fig. 3d. The Sb_2_Se_3_-H photocathode shows an outstanding enhancement in photon-harvesting ability compared to the Sb_2_Se_3_-L photocathode across the entire spectral region. The IPCE value of the Sb_2_Se_3_-L photocathode remains below 68%, while the IPCE value of the Sb_2_Se_3_-H photocathode exceeds 68% across the visible spectrum (400–800 nm) and reaches 91% under 420 nm illumination. The integrated photocurrent density for the Sb_2_Se_3_-L and Sb_2_Se_3_-H devices, calculated from the IPCE curves using the solar AM 1.5 G spectrum, yields 16.9 mA cm^−2^ and 24.7 mA cm^−2^, respectively. Both integrated photocurrents agree well with the values determined from LSV measurements. The photocurrent density of the photoelectrode can be analyzed by eqn (1),

1 *J*_PEC_ = *J*_abs_ × *η*_sep_ × *η*_inj_

where *J*_PEC_ is the measured photocurrent density, *J*_abs_ is the rate of photon absorption expressed as a current density, *η*_sep_ is the charge separation efficiency, and *η*_inj_ is the charge injection efficiency.^42^ Fig. S14† shows the reflectance and absorbance of both Sb_2_Se_3_ samples, indicating almost identical photon absorption efficiency. The value of *η*_inj_ for both Sb_2_Se_3_ photocathodes can be assumed to be equal due to the same Pt co-catalyst loaded by sputtering. Therefore, we identify that the photocurrent enhancement for the Sb_2_Se_3_-H photoelectrode derives from the improved *η*_sep_, *i.e.*, less recombination occurs in the Sb_2_Se_3_-H device.

**Fig. 4:**
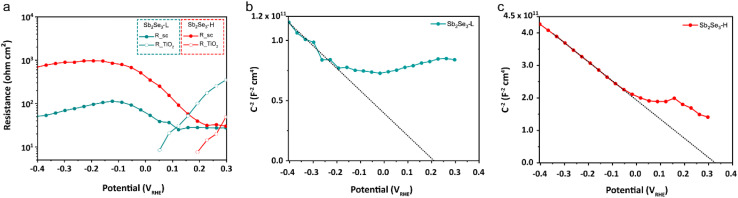
(a) Resistances of the Sb_2_Se_3_-L and Sb_2_Se_3_-H photocathodes from the EIS fitting procedure under 10% white light illumination. Mott–Schottky plots of the (b) Sb_2_Se_3_-L and (c) Sb_2_Se_3_-H photocathodes obtained from EIS fitting under dark conditions.

Electrochemical impedance spectroscopy (EIS) measurements were conducted to characterize the charge recombination of the Sb_2_Se_3_ photocathodes. The Nyquist plots of both photocathodes at potentials ranging from 300 to −400 mV_RHE_ are shown in Fig. S15.† An equivalent circuit (EC) model, consisting of a series resistance and three serially connected RC elements, was employed to fit the Nyquist plot from the EIS measurement, and we identified the RC elements corresponding to the Sb_2_Se_3_ and TiO_2_ layers (see ESI for details†). Our primary focus was on the depletion region at the Sb_2_Se_3_/TiO_2_ interface, as the device structure (FTO/Au/Sb_2_Se_3_-L, Sb_2_Se_3_-H/TiO_2_/Pt) for both photocathodes is identical except for the Sb_2_Se_3_ layer. As shown in Fig. 4, the *R*_SC_ of the Sb_2_Se_3_-H photocathode is one order of magnitude higher than that of the Sb_2_Se_3_-L photocathode, indicating less recombination of photogenerated electron–hole pairs in the Sb_2_Se_3_-H photocathode. Mott–Schottky analysis confirms the p-type nature of both Sb_2_Se_3_-L and Sb_2_Se_3_-H photocathodes. The Sb_2_Se_3_-H photocathode shows a slightly more positive flat band potential (0.34 V_RHE_) compared to the Sb_2_Se_3_-L photocathode (0.22 V_RHE_) in Fig. 4b and c. Moreover, the resistance at the TiO_2_/catalyst interface (*R*_TiO_2__) decreases and vanishes at 0.05 V_RHE_ for the Sb_2_Se_3_-L and 0.2 V_RHE_ for the Sb_2_Se_3_-H as the applied potential approaches the photocurrent onset. The *R*_SC_ of the Sb_2_Se_3_-L and Sb_2_Se_3_ photocathodes starts increasing at 0.1 V_RHE_ and 0.23 V_RHE_, respectively (Table S1†). These findings align well with the results from LSV measurements, where the Sb_2_Se_3_-H photocathodes delivered an earlier onset potential.

**Fig. 5:**
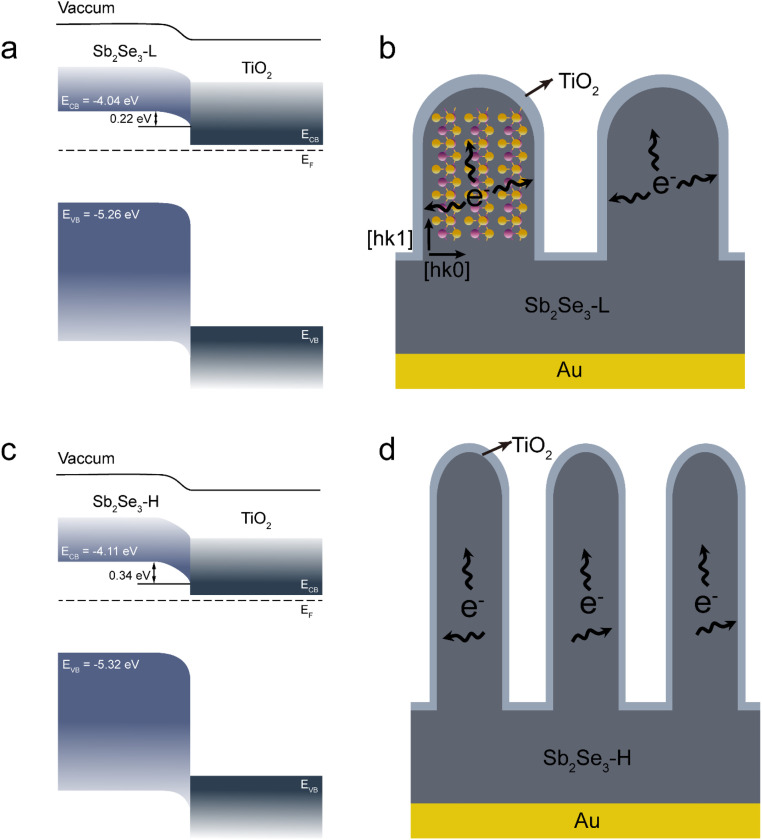
Band diagram of the (a) Sb_2_Se_3_-L and (c) Sb_2_Se_3_-H photocathodes. Schematic illustration of the charge separation mechanism in the (b) Sb_2_Se_3_-L and (d) Sb_2_Se_3_-H photocathodes.

A hypothesis of the charge separation mechanism was proposed to elucidate the PEC performance enhancement in the Sb_2_Se_3_-H photoelectrode. Derived from the IPCE measurements, the optical band gaps for both Sb_2_Se_3_ films were determined to be the same (around 1.21 eV, Fig. S16†).^43^ The Fermi level (*E*_F_) position of both Sb_2_Se_3_ films can be determined by the flat band potential. The *E*_F_ position with respect to the valence band maximum (*i.e.*, the doping level) was estimated using the XPS valence band maxima (Fig. S17†). The band gap of TiO_2_ was determined by Tauc plot by measuring the transmittance of the TiO_2_ film (Fig. S18†). With these values, the energy band alignments of the Sb_2_Se_3_/TiO_2_ heterojunction for both devices were constructed before contact, as seen in Fig. S19.† The energy band bending of the Sb_2_Se_3_/TiO_2_ heterojunction obtained after *E*_F_ equilibration is presented in Fig. 5a and c. It revealed that a slightly larger band bending formed in the Sb_2_Se_3_-H photocathode compared with the Sb_2_Se_3_-L photocathode. This indicates that photoexcited electrons can be more efficiently extracted in the Sb_2_Se_3_-H photocathode at the same biased potential. In addition, the crystal structure has negligible influence on the PEC performance since both Sb_2_Se_3_ samples have identical crystal orientations. However, the morphology displays a big difference between the Sb_2_Se_3_-H and Sb_2_Se_3_-L samples. For the Sb_2_Se_3_-H thin film, it has longer and thinner nanorods compared to the Sb_2_Se_3_-L thin film. Due to the anisotropic properties of Sb_2_Se_3_, electron mobilities vary, making it easier for photoexcited electrons to transport along (Sb_4_Se_6_)_n_ ribbons connected by covalent bonds, while it is more difficult to cross ribbons bonded by van der Waals forces.^18^ Based on these morphological differences and the anisotropy in charge carrier mobility, a charge transport mechanism was proposed. The photoexcited electrons in the Sb_2_Se_3_ bulk must be transported to the TiO_2_/electrolyte surface through the nanorods or the valleys among the nanorods where they undergo the hydrogen evolution reaction. When the photoexcited electrons are transported to the vertically oriented nanorods, they can be extracted along either the [*hk*1] or the [*hk*0] orientations, as illustrated in Fig. 5b and d. The reduced diameter of the nanorods in the Sb_2_Se_3_-H thin film is advantageous for charge transport due to the decreased charge transport lengths in the [*hk*0] direction, enhancing the overall PEC performance. Additionally, Sb_2_O_3_ typically forms at areas with dangling bonds, such as the tops of nanorods or valleys in our samples, and is known to act as a recombination center for electron–hole pairs. As previously discussed, the (NH_4_)_2_S etching treatment can improve the performance of Sb_2_Se_3_-based photocathodes and solar cells by removing the Sb_2_O_3_ layer. However, in our case, the PEC performance of both untreated and etched Sb_2_Se_3_ photocathodes (Fig. S13†) remained identical. Based on this observation, we propose that photon-generated electrons are primarily extracted along the sides of the nanorods, in the [*hk*0] direction, which are free of dangling bonds. This result further supports our hypothesis.

## 3. Conclusion

We demonstrated a facile method to enhance the PEC performance of Sb_2_Se_3_ photocathodes by varying the substrate temperature during Sb metal deposition, thereby exposing different Sb surface planes and affecting the resulting Sb_2_Se_3_ morphology. The champion Sb_2_Se_3_-H photocathode exhibited a higher photocurrent density of −22.1 mA cm^−2^ at 0 V_RHE_ and an onset potential of 0.31 V_RHE_, compared with the champion Sb_2_Se_3_-L photocathode, which showed a photocurrent density of −15.2 mA cm^−2^ at 0 V_RHE_ and an onset potential of 0.21 V_RHE_. The improved PEC performance of the Sb_2_Se_3_-H photocathode is primarily attributed to the reduced charge recombination and the difference in flat band potential. We proposed that the charge transport mechanism in the thinner vertically oriented nanorods in the Sb_2_Se_3_-H photocathode facilitate separation and transport in the horizontal [*hk*0] direction of the nanorods, thereby enhancing the overall PEC performance.

## 4. Experimental section

### 4.1. Preparation of Sb_2_Se_3_ films

Sb powder (99.999%, Kurt J. Lesker Co. Ltd) was used as the evaporation source to deposit Sb thin films on FTO/Au substrates *via* thermal evaporation using the VapourPhase/PicoSphere system (Oxford Vacuum Science Ltd). Before deposition, FTO glass (FTO TEC 15, Pilkington, Tokyo, Japan) was cleaned sequentially with acetone, a 5% deconex solution in water, distilled water, and isopropanol in an ultrasonic bath for 10 min each, followed by drying under a stream of N_2_. After cleaning, a 150 nm Au layer with a 10 nm Cr adhesion layer was deposited by sputtering (Safematic CCU-010 sputter coater). Sb layers (240 nm) were evaporated at substrate temperatures of 25 °C and 75 °C with a deposition rate of 0.1 nm s^−1^ under a pressure of 1 × 10^−5^ Pa. The substrate temperature was controlled using a Sub-θ rotary stage and NanoSphere software, ensuring consistent thermal conditions during deposition. The evaporation source was positioned 35 cm from the sample holder. Subsequently, selenium powder (70 mg) and the as-prepared Sb metal films were placed in separate zones of a two-zone tube furnace (Kejia Co. Ltd). The Sb metal films were selenized at 325 °C for 30 min with a ramping time of 20 min under Ar protection. Once the process was finished, the tube furnace was opened to cool down to room temperature within 60 min. The nanorod diameters were measured using ImageJ software. We first calibrated the scale using the scale bar in the SEM images. To ensure accuracy, the diameters were then measured manually at the end of the nanorods. To minimize the effect of the inclination, the measurement direction is typically perpendicular to the tilt direction of the nanorods. For the etching treatment, Sb_2_Se_3_-H thin films were immersed in a diluted (NH_4_)_2_S solution (Sigma-Aldrich, 40–48 wt% in H_2_O) (10 ml, 10–12 wt%) at room temperature for 5 s, then rinsed with distilled water for 10 s and dried under a flow of N_2_.

### 4.2. Deposition of TiO_2_ layer and Pt catalyst

TiO_2_ was deposited using atomic layer deposition (ALD) with a Picosun R200 system. The tetrakis (dimenthylamido) titanium(iv) (TDMAT) (99.999%, Sigma-Aldrich, Buchs, Switzerland), and Milli-Q water were used as the Ti and O precursors, respectively. The titanium precursor was heated to 85 °C, and the reactor chamber was maintained at 120 °C during deposition. For the preparation of Sb_2_Se_3_ photocathodes, 930 cycles were performed to achieve a 50 nm TiO_2_ layer. A small piece of the silicon wafer was placed beside the samples to check the film's thickness by ellipsometry. The Pt cocatalyst was sputtered onto the surface of the as-fabricated Sb_2_Se_3_/TiO_2_ electrodes using a sputter coater (Safematic CCU-010). The distance between the target and the samples was set to 5 cm. The chamber was purged three times before sputtering. A nominal 2 nm Pt layer was sputtered under an applied current of 10 mA for 100 s in an Ar atmosphere.

### 4.3. Materials characterization

The morphologies of metallic Sb films and Sb_2_Se_3_ films were characterized using SEM (Zeiss Gemini 450). XRD (Rigaku SmartLab) with Cu Kα radiation (*λ* = 0.15406 nm) was employed to characterize the crystal structures of Sb metal and Sb_2_Se_3_ films. UV-vis spectra were recorded on a Shimadzu UV-3600 spectrometer in an integrating sphere. The absorbance and reflectance spectra of the Sb_2_Se_3_ devices were determined. XPS analysis was conducted using a physical electronics Quantum 2000 X-ray photoelectron spectrometer equipped with monochromatic Al Kα radiation, operating at 15 kV and 32.3 W. To ensure accurate measurements, the instrument's energy scale was calibrated using an Au reference sample. The analysis was performed under a vacuum level of 1 × 10^−6^ Pa, with an electron take-off angle of 45° and a pass energy of 23.5 eV. Shirley background subtraction was employed with instrument-specific sensitivity factors. Core-level spectra were meticulously deconvoluted to discern contributions from multiple elements, utilizing a GL 30 asymmetric line shape. Notably, a Δ*E* of 9.34 eV for the Sb 3d doublet was applied for accurate deconvolution of the spectra.

### 4.4. PEC performance and EIS measurements

PEC performance measurements of the Sb_2_Se_3_ photocathodes were carried out in a typical three-electrode cell configuration, with a Pt wire serving as the counter electrode and an Ag/AgCl electrode (saturated solution of KCl) as the reference electrode. A potentiostat (a Bio-Logic Sp-200) was used to control the potential of the working electrode. The Sb_2_Se_3_ photocathodes were evaluated in a 1 M H_2_SO_4_ solution and irradiated with simulated AM 1.5 G illumination from a Xenon lamp, calibrated to 100 mW cm^−2^ with a silicon diode from PV measurements, Inc (PVM558). The area of the photocathode was defined with epoxy resin, and the active area was measured by counting pixels using the freeware image processing software Gimp. IPCE spectra of the Sb_2_Se_3_ photocathodes were obtained using a home-built system under monochromatic light irradiation at 0 V_RHE_ under 10% white light illumination. The photon flux at each wavelength was first calibrated with a Si photodiode before the measurement of the sample. The IPCE was calculated by the following equation:2
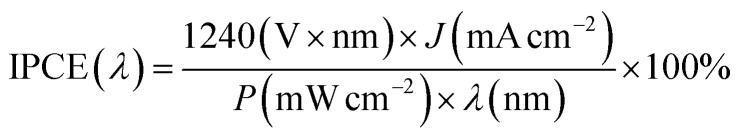
where *J* is the photocurrent density, *P* is the light intensity at each wavelength, *λ* is the wavelength of the monochromatic light.

EIS measurements were also performed using the same potentiostat combined with a frequency analyzer. A 10% white light illumination from LEDs (SP-12-W5, cool white Luxeon Rebel) was applied for the investigations. The light intensity was calibrated with a calibrated silicon diode with a BK7 window. To minimize the formation and release of gas bubbles, TritonX (1 mM) was added to the H_2_SO_4_ electrolyte solution. The DC potential ranged from 300 mV to −400 mV_RHE_ and was scanned in 35 mV steps, with a modulation voltage of *V*_rms_ = 10 mV. The frequency range applied was 7 MHz to 0.2 Hz, and the EIS spectra were analyzed using ZView software from Scribner.

## Data availability

The data supporting this article have been included as part of the ESI.†

## Author contributions

S. D. T. and Z. W. designed the experiment. W. N. provided supervision and advice. Z. W. and Y. G. fabricated and tested all the devices. E. S. and T. M. performed the EIS measurements. P. A. carried out the XPS measurement.

## Conflicts of interest

The authors declare no competing financial interests.

## Supplementary Material

TA-013-D4TA07389D-s001
