# Supplementary material for: Nanowire morphology control in Sb metal-derived antimony selenide photocathodes for solar water splitting
Source: J Mater Chem A Mater. 2025 Feb 17;13(12):8416–24. doi: 10.1039/d4ta07389d (PMC11848248; doi:10.1039/d4ta07389d)
Supplement: TA-013-D4TA07389D-s001 [file TA-013-D4TA07389D-s001.pdf]

Supplementary Information (SI) for Journal of Materials Chemistry A.
This journal is © The Royal Society of Chemistry 2025

Supporting information

Nanowire Morphology Control in Sb Metal-derived Antimony Selenide Photocathodes for Solar Water Splitting

Zhenbin Wang, Yongping Gan, Dr. Erin Service, Dr. Pardis Adams, Dr. Thomas Moehl, Dr. Wenzhe Niu*, Prof. Dr. S. David Tilley*

Department of Chemistry, University of Zurich, Winterthurerstrasse 190, 8057 Zurich, Switzerland.

Dr. Wenzhe Niu
Laboratory of Photonics and Interfaces, Institute of Chemical Sciences and Engineering, École Polytechnique Fédérale de Lausanne, Lausanne 1015, Switzerland

E-mail: wenzhe.niu@epfl.ch
E-mail: david.tilley@chem.uzh.ch

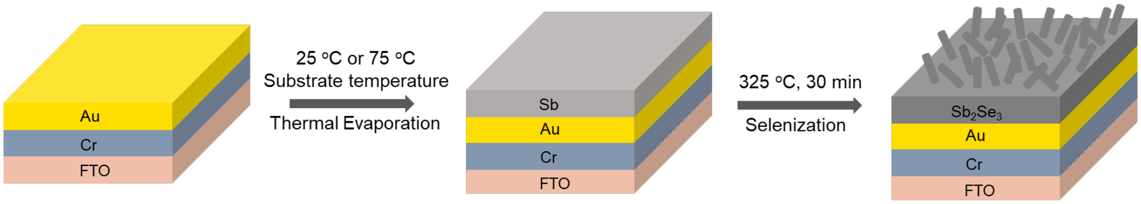

Figure S1. Schematic illustration showing the fabrication process of the nanostructured Sb_2Se_3 thin films.

Various selenization conditions, including temperature, duration, and Se vapor pressure, were systematically investigated during the early stages of this research. Among these, a selenization condition of 325 °C for 30 minutes was found to deliver the best PEC performance for the Sb_2Se_3 photocathode and was subsequently adopted for this study.

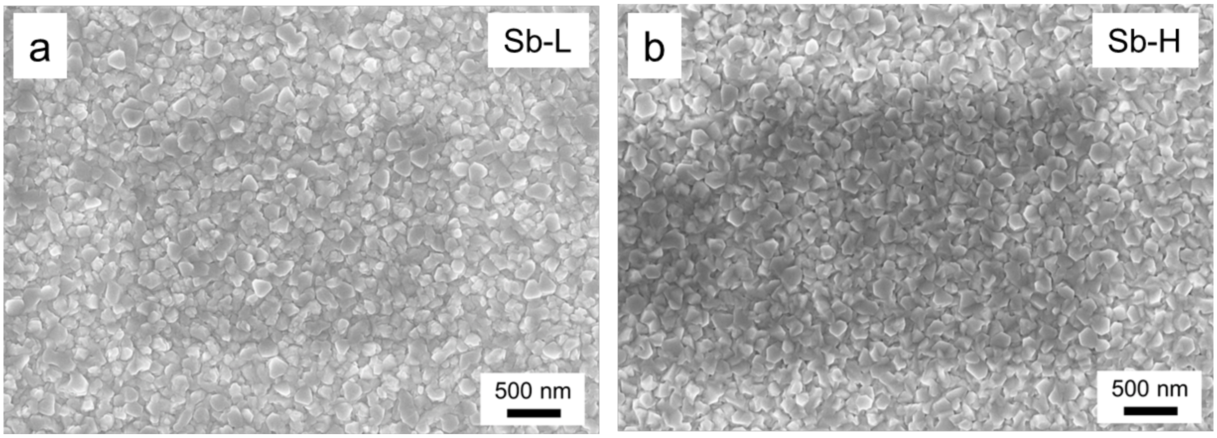

Figure S2. Top-view SEM images of the (a) Sb-L and (b) Sb-H thin films evaporated at substrate temperatures of 25 °C and 75 °C.

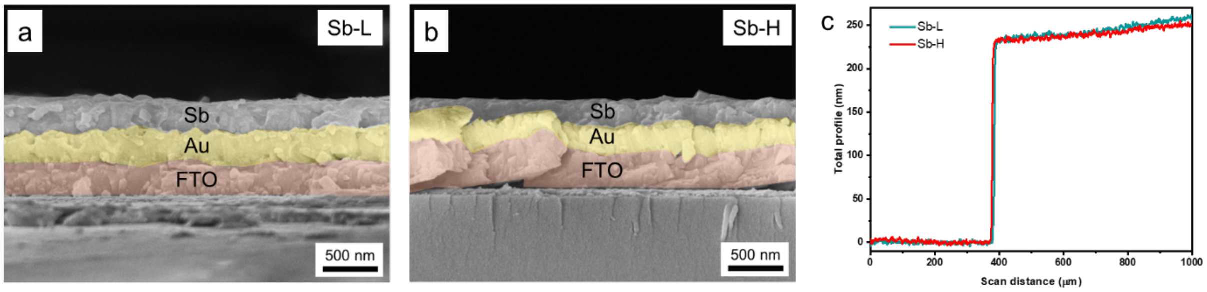

Figure S3. Cross-sectional SEM images of the (a) Sb-L and (b) Sb-H thin films. (c) The thickness of the metallic Sb thin films determined by a profilometer.

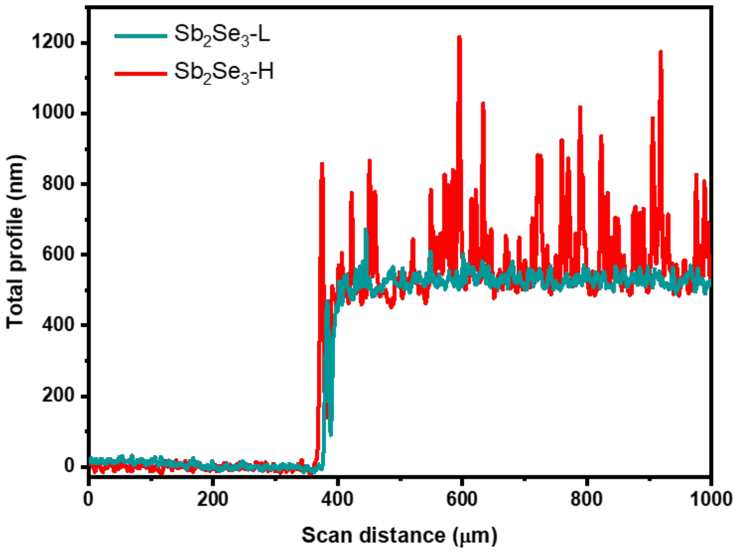

Figure S4. The thickness of the Sb_2Se_3 thin films determined by a profilometer.

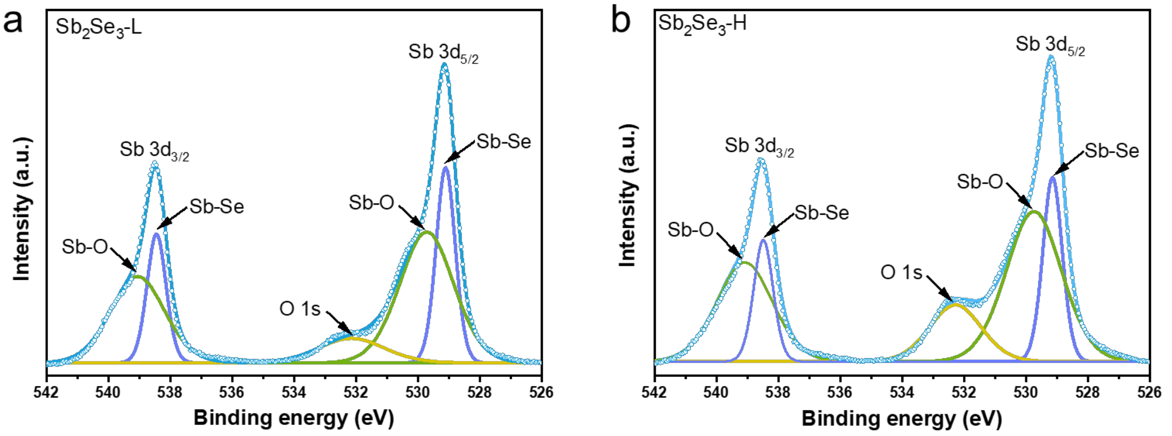

Figure S5. Sb 3d XPS spectra of (a) the Sb_2Se_3-L and (b) Sb_2Se_3-H thin films.

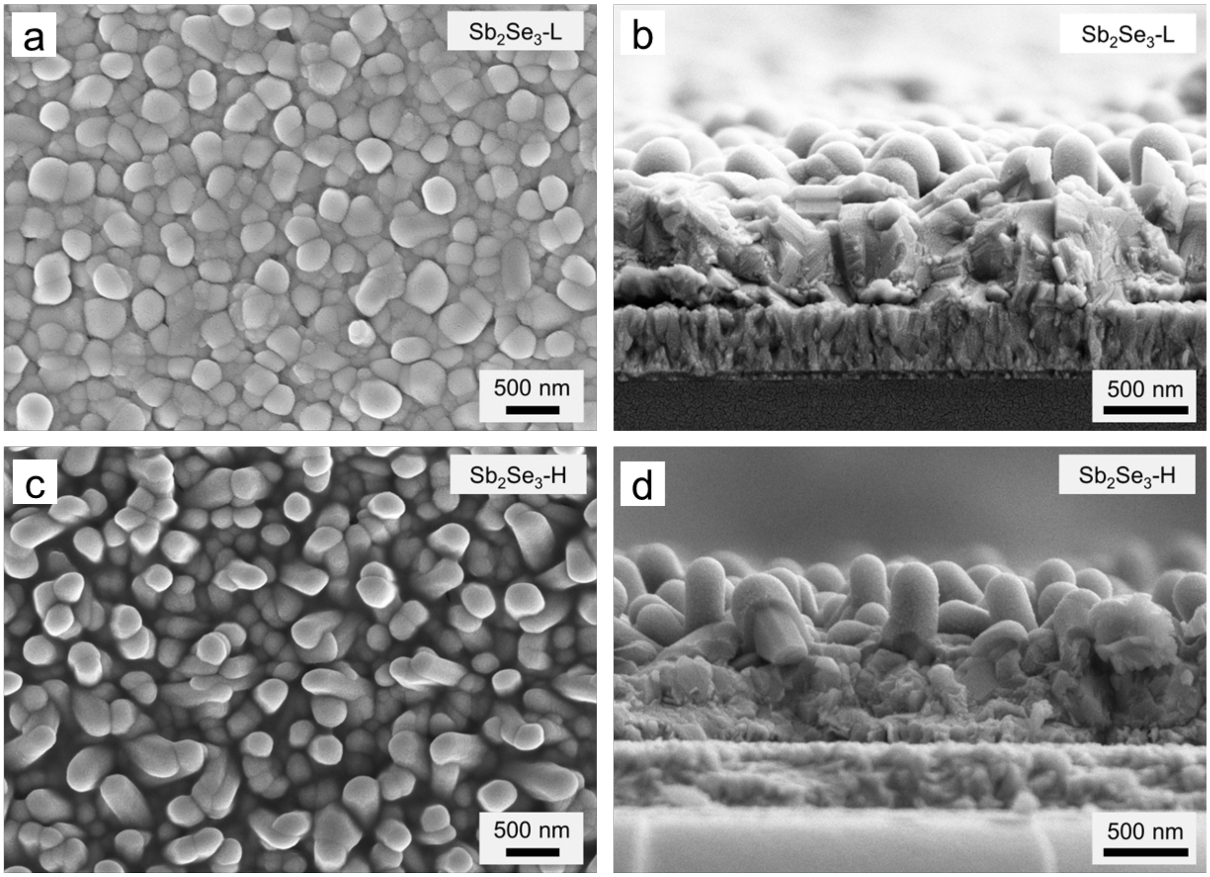

Figure S6. Top-view and cross-sectional SEM images of the (a, b) FTO/Au/Sb_2Se_3-L/TiO_2/Pt and (c, d) FTO/Au/Sb_2Se_3-H/TiO_2/Pt thin films.

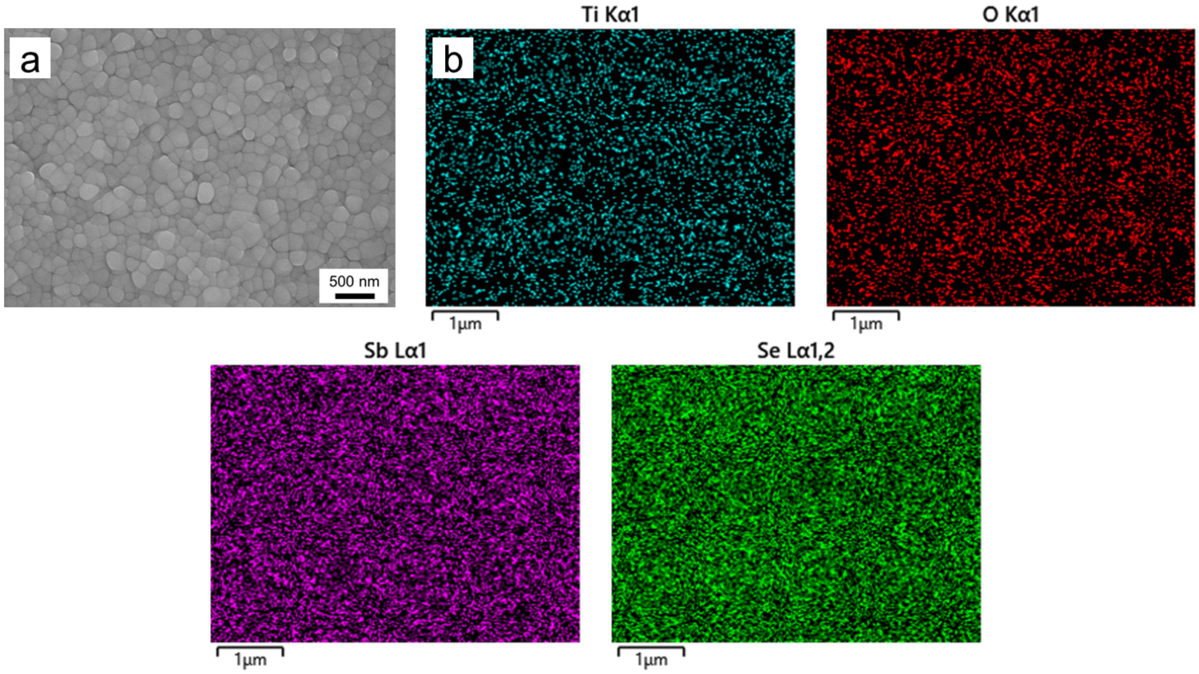

Figure S7. (a) Top-view SEM image and (b) the corresponding EDS spectra of the Sb_2Se_3 thin film covered with TiO_2.

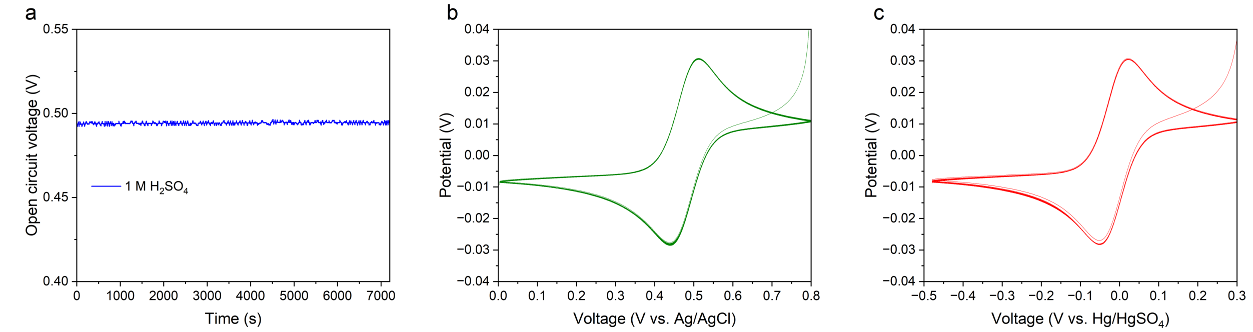

Figure S8. (a) Open-circuit voltage (OCV) measurements conducted in 1 M H_2SO_4, with $Hg/HgSO_4$ (1 M H_2SO_4) and Ag/AgCl (3 M KCl) serving as the working and counter electrodes, respectively. Cyclic voltammetry (CV) measurements over 1.5 hours for the Fe^{3+}/Fe^{2+} redox couple in 1 M H_2SO_4, using (b) Ag/AgCl (3 M KCl) and (c) $Hg/HgSO_4$ (1 M H_2SO_4) as reference electrodes.

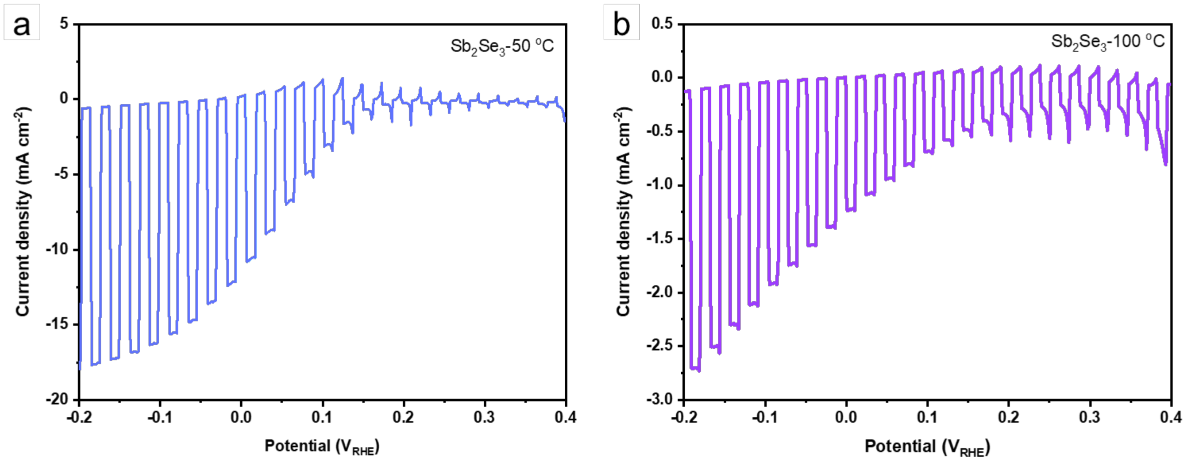

Figure S9. LSV measurements of the Sb_2Se_3-50 ºC and Sb_2Se_3-100 ºC photocathodes under intermittent illumination (simulated AM 1.5 G, 100 mW cm^{-2}) in a 1 M H_2SO_4 electrolyte with a scan rate of 10 mV s^{-1}.

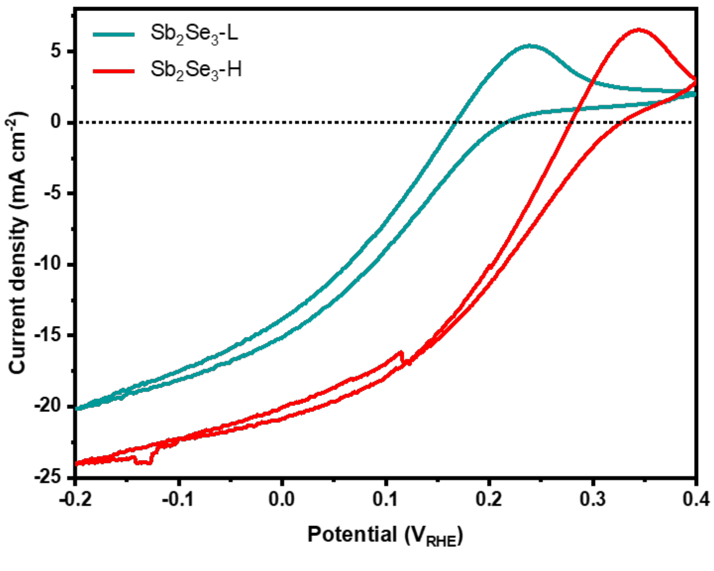

Figure S10. Cyclic voltammetry (CV) measurements of the Sb_2Se_3-L and Sb_2Se_3-H photocathodes under illumination (simulated AM 1.5 G, 100 mW cm^{-2}) in a 1 M H_2SO_4 electrolyte with a scan rate of 10 mV s^{-1}.

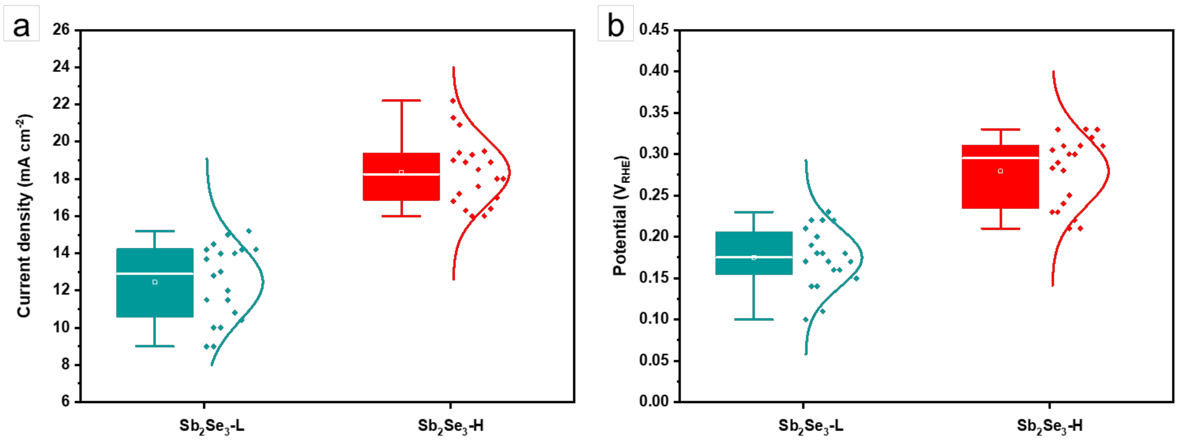

Figure S11. Box plots of (a) short circuit current density and (b) onset potential achieved from 20 samples of each category.

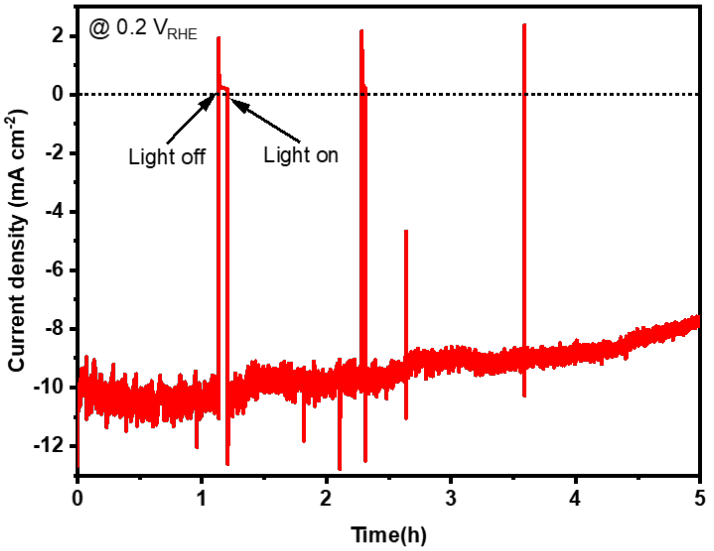

Figure S12. Stability test of the Sb_2Se_3-H photocathode at 0.2 V_{RHE} under AM 1.5 G simulated solar illumination (100 mW cm^{-2}) in a 1 M H_2SO_4 electrolyte solution.

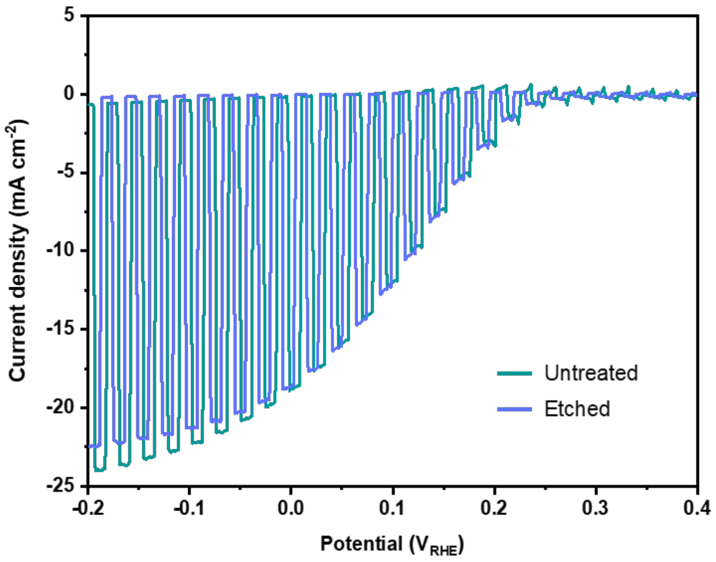

Figure S13. LSV measurements of Sb_2Se_3-H photocathodes with and without $(NH_4)_2S$ etching treatment under intermittent illumination (simulated AM 1.5 G, 100 mW cm^{-2}) in a 1 M H_2SO_4 electrolyte with a scan rate of 10 mV s^{-1}.

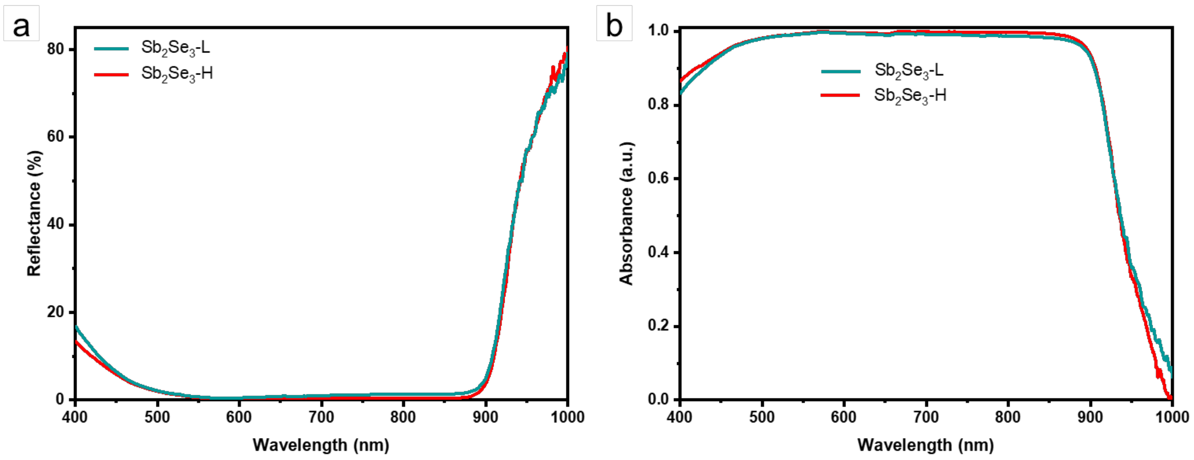

Figure S14. (a) The reflectance spectrum of the FTO/Au/Sb_2Se_3-L/TiO_2/Pt and FTO/Au/Sb_2Se_3-H/TiO_2/Pt devices. (b) The absorbance spectrum of the FTO/Sb_2Se_3-L/TiO_2/Pt and FTO/Sb_2Se_3-H/TiO_2/Pt devices.

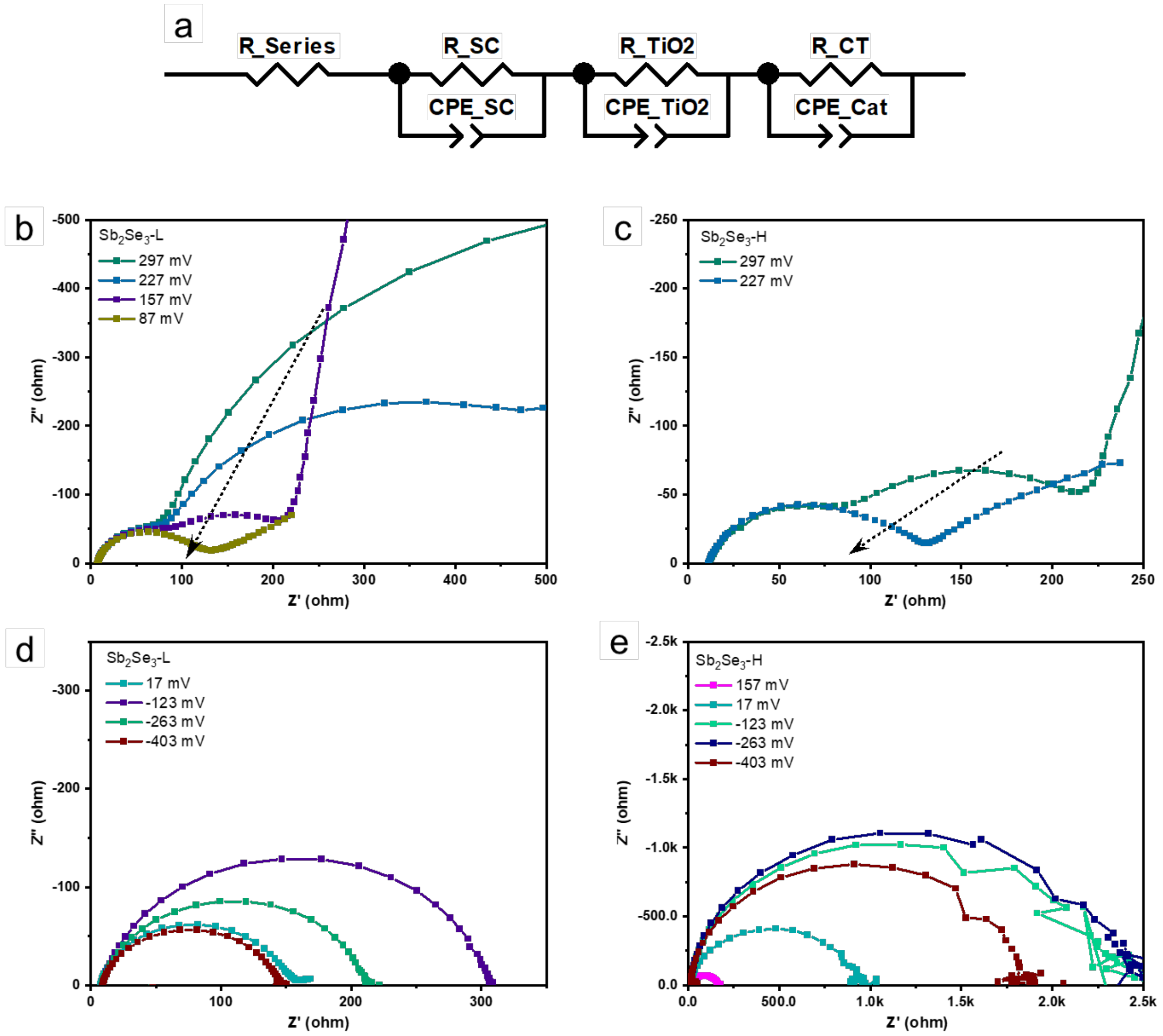

Figure S15. (a) The equivalent circuit is used for the EIS fitting. Nyquist plots of (b) the Sb_2Se_3-L and (c) Sb_2Se_3-H photocathodes before onset potential under 10% white light illumination. Nyquist plots of (d) the Sb_2Se_3-L and (e) Sb_2Se_3-H photocathodes after onset potentials under 10% white light illumination.

The Nyquist plots at different applied potentials are shown in Figure S9. The equivalent circuit model (Figure S9a) was used for the EIS fitting. To improve the fitting accuracy, a constant phase element (CPE) was utilized instead of an ideal capacitor. The Nyquist plots at different applied potentials are shown in Figure S9. The equivalent circuit model (Figure S9a) was used for the EIS fitting. To improve the fitting accuracy, a constant phase element (CPE) was utilized instead of an ideal capacitor. At potentials positive of photocurrent onset, three elements are observed (Figure

S9b,c): a high-frequency element corresponding to the semiconductor, a mid-frequency element corresponding to the TiO_2, and a low frequency element corresponding to the catalyst. The resistances corresponding to the TiO_2 and catalyst decrease as the potential moves towards more negative values. As the onset potential is reached, they then become negligible, indicating that thermodynamic barriers to photogenerated charge transfer have been overcome. After the onset potential, only the element corresponding to the semiconductor remains. This element increases in size, such that the capacitance corresponds to the space charge capacitance, and the resistance corresponds to the inverse slope of the JV curve at that potential (the DC resistance).

Table S1. Extracted R_{SC} values of the Sb_2Se_3-L and Sb_2Se_3-H photocathodes from the EIS fitting procedure under 10% white light illumination.

Voltage (V_{RHE})	Sb_2Se_3-L (ohm cm^2)	Sb_2Se_3-H (ohm cm^2)
0.297	46.702	30.229
0.262	46.512	32.6306
0.227	44.65	31.4792
0.192	43.092	39.406
0.157	41.192	57.722
0.122	39.178	91.998
0.087	37.4946	154.622
0.052	39.862	252.852
0.017	50.426	348.27
-0.018	68.742	513.76
-0.053	91.884	679.44
-0.088	113.62	797.24
-0.123	120.688	854.62
-0.158	113.164	961.78
-0.193	109.744	968.24
-0.228	87.552	967.1
-0.263	77.9	898.32
-0.298	70.984	901.74
-0.333	58.596	840.56
-0.368	51.262	772.16
-0.403	50.502	694.26

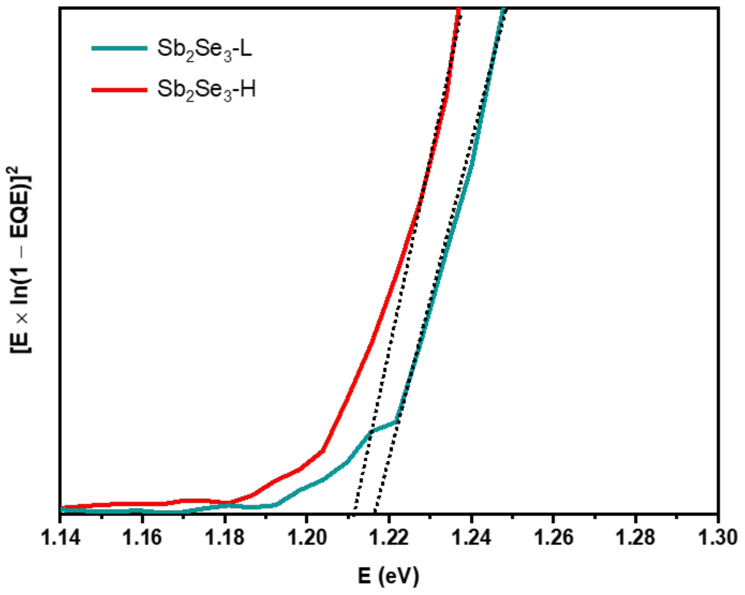

Figure S16. The bandgap of the Sb_2Se_3-L and Sb_2Se_3-H samples determined from IPCE measurements.

For an ideal junction, the external quantum efficiency (EQE) can be approximated by the following equation:[1]

$$EQE = 1 - \frac{\exp(-\alpha W)}{1 + \alpha L_n} \ (1)$$

where α is the absorption coefficient, W is the width of the space charge region and L_n is the minority carrier diffusion length. For $\alpha L_n < 1$, implying a very short L_n, Equation (1) simplifies to:

$$EQE = 1 - \exp(-\alpha W) \ (2)$$

For indirect transitions, the dependence of absorption coefficient on photon energy is described by:

$$\alpha h v \propto (h v - E_g)^2 \ (3)$$

Thus, a plot of $[E \ x \ (E - E_g)]^2$ against E can be used to extract the band gap E_g.

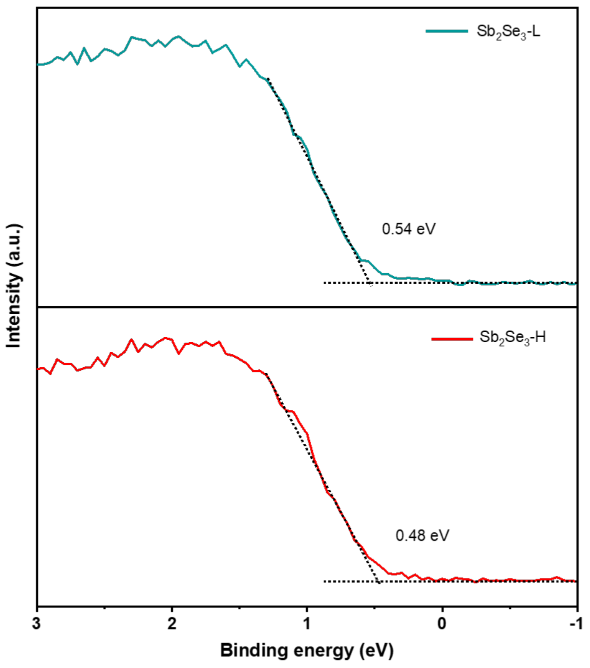

Figure S17. XPS measurements of the valence band edge of Sb_2Se_3-L and Sb_2Se_3-H thin films.

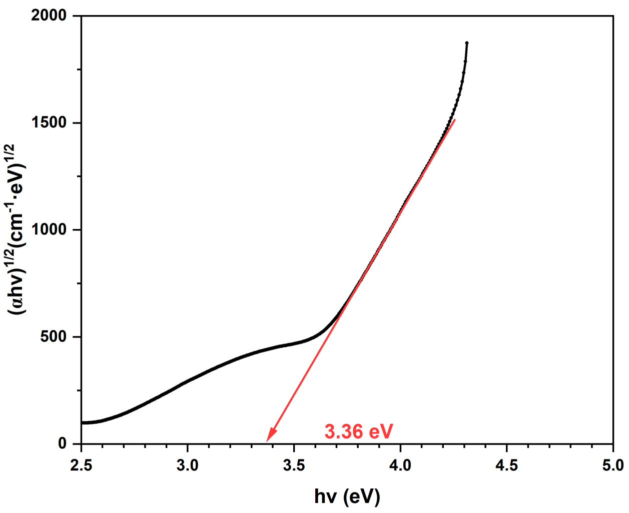

Figure S18. The Tauc plot derived from the transmittance data of a 100 nm thick TiO_2 film deposited on glass substrate by ALD.

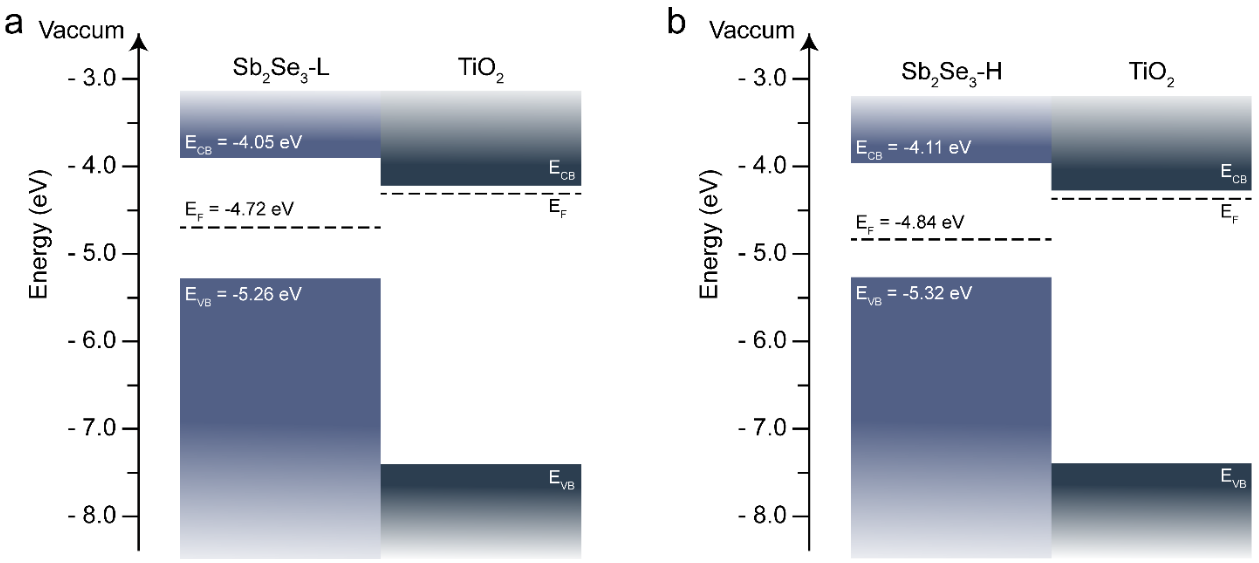

Figure S19. (a) Band alignments of the (a) Sb_2Se_3-L/TiO_2 and (c) Sb_2Se_3-H/TiO_2 devices.

The bandgaps of the Sb_2Se_3-L and Sb_2Se_3-H devices were determined to be 1.17 eV using the Kubelka-Munk function. The Fermi level (E_F) positions of both devices with respect to the vacuum level were determined by the flat band potential. The flat band potentials obtained from Mott-Schottky plots indicate the difference between the Fermi levels of Sb_2Se_3 and the redox potential of the H_2SO_4 electrolyte solution (pH 0, $E_{H+/H2}$, 0 V_{RHE}). The work functions of Sb_2Se_3-L and Sb_2Se_3-H were calculated to yield values of 4.72 V and 4.84 V, respectively. XPS measurements revealed energetic distances from the E_F to the valence band maximum (VBM) of 0.54 eV for Sb_2Se_3-L and 0.48 eV for Sb_2Se_3-H. Based on these measurements, the VBM positions were determined to be 5.26 eV for Sb_2Se_3-L and 5.32 eV for Sb_2Se_3-H, while the conduction band minimum (CBM) positions were calculated to be 4.09 eV for Sb_2Se_3-L and 4.15 eV for Sb_2Se_3-H. According to this, the band alignments of the Sb_2Se_3-L/TiO_2 and Sb_2Se_3-H/TiO_2 interfaces were constructed.

References

1 G. Zoppi, I. Forbes, R. W. Miles, P. J. Dale, J. J. Scragg and L. M. Peter, *Progress in Photovoltaics: Research and Applications*, 2009, **17**, 315–319.
